# Surgical Outcomes of Synchronous Multiple Primary Non-Small Cell Lung Cancers

**DOI:** 10.1038/srep23252

**Published:** 2016-06-02

**Authors:** Zhirong Zhang, Shugeng Gao, Yousheng Mao, Juwei Mu, Qi Xue, Xiaoli Feng, Jie He

**Affiliations:** 1 Department of Thoracic Surgery, Cancer Hospital of Chinese Academy of Medical Sciences and Peking Union Medical College, China; 2 Department of Pathology, Cancer Hospital of Chinese Academy of Medical Sciences and Peking Union Medical College, China

## Abstract

The prognostic indicators for synchronous multiple primary non-small cell lung cancer (NSCLC) vary across reports. In present study, the prognostic factors for the patients with synchronous multiple primary NSCLC were analyzed in a large cohort. A total of 285 patients with synchronous multiple primary NSCLC who underwent radical surgical resection and with complete follow-up information were included in this study. The Kaplan-Meier method were used for survival analysis, Cox proportional hazards regression models were used for risk factors evaluation. Among them, 94 (33.0%) patients had bilateral tumors and 51 (17.9%) had multiple (≥3) tumors. The 5-year disease-free survival (DFS) and overall survival (OS) rate was 58.7% and 77.6%, respectively. Univariate analysis identified parameters conferring shorter OS including male gender, symptomatic disease, negative family history, large maximal tumor size, not all adenocarcinomas, advanced highest T stage, and lymph node involvement. Multivariate analysis showed that male gender (p = 0.020), symptomatic disease (p = 0.017), and lymph node involvement (p < 0.001) were independent adverse prognosticators. For patients with multiple adenocarcinomas, the 5-year DFS and OS rate was 59.6% and 82.4%, respectively. The subtypes other than lepidic predominant (p < 0.001) and lymph node involvement (p = 0.002) were the independent unfavorable prognosticators. In conclusion, we identified independent prognosticators which will provide the valuable clues for postoperative management of patients with synchronous multiple primary NSCLC.

According to the current TNM classification system (the 7th version) for lung cancer^1^, multiple tumor nodules in the same lobe are classified as T3, and if multiple tumor nodules are located on the same side but in a different lobe or on the contralateral side, the tumors are categorized as T4 or M1a. In proposals suggesting revisions to T descriptors in the forthcoming 8th edition of the TNM classification^2^, the above definitions have not been changed. However, these categories are based on the assumption that multiple nodes are intrapulmonary metastases that mainly originate from the primary lung cancers^3^.

In clinical practice, a large number of multiple tumor nodules are now demonstrated as synchronous multiple primary lung cancers (SMPLCs) as a result of the worldwide use of high resolution imaging systems. It is of immense clinical importance that rigorous clinical or histopathological criteria enable to distinguish SMPLC from intrapulmonary metastatic diseases, which significantly influences staging, therapeutic strategies and long-term survival of lung cancer. In 1975, Martini and Melamed initially proposed diagnostic criteria to discriminate synchronous and metachronous multiple lung cancers from intrapulmonary metastases in 50 patients^4^. This diagnostic algorithm was then modified and optimized as more information, including genetic and molecular analyses, became available, and have improved clinical accuracy and mitigated the problems of differential diagnosis.

As reported in previous clinical series, the incidence rate of SMPLC varied from 0.2% to 8% (3.5% to 14% in autopsy studies)^5^, and the 5-year overall survival (OS) rate for SMPLC ranged from 0% to 82%^6^,^7^, resulting from differences in inclusion criteria, patient’s baseline characteristics or the sample size of patient population. Hence, the prognostic factors associated with prolonged survival differ between studies, and it is difficult to draw solid conclusions that can be widely used to evaluate prognoses in patients with SMPLC.

In a large cohort of patients, we analyzed surgical outcomes of synchronous multiple primary non-small cell lung cancer (NSCLC) to investigate the prognostic values of various clinical parameters for long-term survival. The present study, to our knowledge, is the largest investigation on clinical outcome of patients treated with surgery for synchronous multiple primary NSCLC.

## Materials and Methods

### Information collection

The medical records of patients who underwent complete pulmonary resection for lung cancer from January 2010 to December 2014 at the Department of Thoracic surgery, Cancer Hospital of Chinese Academy of Medical Sciences were reviewed. The demographic characteristics were recorded for further analysis, including: age, gender, major complaint (symptomatic disease was defined as persistent symptoms such as dry cough prior to diagnosis; asymptomatic disease was defined as lung cancers identified by either health check-up and screening or incidental discovery without any symptoms), smoking (never smokers were defined as consumption of <100 cigarettes during their lifetimes; light smokers, consumption of <20 pack-years; moderate smokers, consumption of 20–40 pack-years; and heavy smokers, consumption of >40 pack-years), family history of cancer (in first degree relatives), preoperative serum biomarker profiling (carcinoembryonie antigen [CEA], cancer antigen 125 [CA125], cytokeratin 19 fragments [CYFRA 21-1], squamous cell carcinoma antigen [SCCA] and neuron specific enolase [NSE]), type of surgical resection, as well as the numbers, location (laterality and lobe), size (maximum diameter), and histological type of tumors, the highest T and N stage of each patient, and postoperative adjuvant chemotherapy. Informed consents were signed by all patients. This study was approved by the Institutional Review Board of Cancer Hospital of Chinese Academy of Medical Sciences and conducted according to the guidelines approved by the ethics committee.

### Patient selection

The synchronous preoperative computed tomography (CT), positron emission tomography (PET) and the intra- and postoperative histopathologic diagnoses were used to verify the existence of more than one malignant tumors. Patients were classified with SMPLCs if they met the modified criteria of Martini and Melamed for the diagnosis^8^: (1) Tumors with different histopathologic characteristics (e.g., adenocarcinoma vs. squamous cell carcinoma); (2) Tumors with differently predominant histologic subtypes (e.g., ratio of acinar, bronchoalveolar, and papillary percentage for adenocarcinomas); (3) Tumors with similar histology a. arising from separate foci (e.g., in the case of squamous cell carcinoma, the presence of *in situ* carcinoma in both tumors); b. without regional or mediastinal lymph node metastasis; c. without distant metastasis. Patients with small cell lung cancer, atypical adenomatous hyperplasia or preoperative neoadjuvant therapy were excluded.

### Preoperative evaluation and Surgical approach

The routine preoperative staging workup for lung cancer patients in our hospital includes chest radiography, computed tomography (CT) of the thorax, abdominal ultrasonography, bronchoscopy, magnetic resonance image (MRI) of the brain, whole body bone scan, and cardiopulmonary function test. Endobronchial ultrasonography-guided transbronchial needle aspiration or, positron emission tomography (PET) CT scan was used in patients who had suspected mediastinal lymph nodes. A curative operation for pulmonary resection and lymphadenectomy via thoracotomy or video-assisted thoracic surgery (VATS) were performed.

### Pathology examination

For adenocarcinomas, the tumors were reviewed by a specialized thoracic pathologist (Dr. Feng), and further classified according to the International Association for the Study of Lung Cancer (IASLC) classification^9^ and predominantly histological subtype of largest tumor was recorded for each patient.

### Follow-up

Postoperative surveillance was scheduled regularly in the outpatient clinic with chest CT, serum tumor markers measurement (CEA, CA125, CYFRA 21-1, SCCA and NSE), and abdominal ultrasonography. Bone scanning and MRI of brain were performed annually. The follow-up frequency was every three months for the first 2 years, every six months for the third year, and once per year for subsequent years.

### Statistical analysis

Disease-free survival (DFS) was calculated from the date of the initial surgery to the date of recurrence or distant metastasis; and overall survival (OS) was calculated from the date of the initial surgery to the date of cancer-related death or last follow-up for censored patients. One patient was lost to follow-up. The latest follow-up date was August 12, 2015. Patients who died from cardiopulmonary complications and non-cancer-related reasons were not included in the survival analysis.

Continuous variables were summarized as mean ± standard deviation (SD). Actuarial survival was estimated using Kaplan-Meier method, and log-rank tests were used for univariate analysis. Multivariate analyses were performed using the Cox proportional hazard models to determine prognostic parameters associated with survival. All data analyses were performed using SPSS software version 20.0, and a *P* value of less than 0.05 was considered as statistically significant.

## Results

### Demographics of SMPLC patients

**Figure 1:**
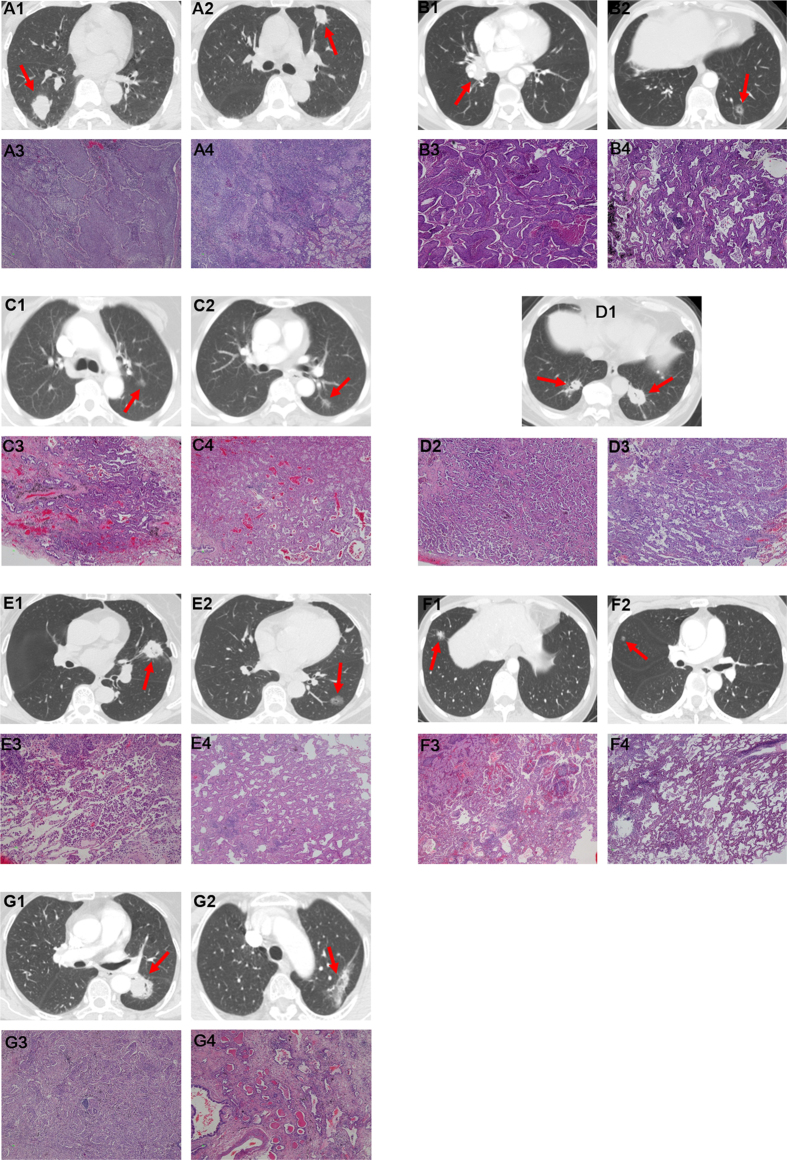
Synchronous multiple primary lung cancers were detected on computed tomography (CT) and confirmed by pathological diagnoses (Hematoxylin-eosin staining, 40×). (**A**) Squamous cell carcinoma in the right lower lobe (**A1,A3**) and left upper lobe (**A2,A4**) of the lung. (**B**) Squamous cell carcinoma in the right lower lobe (**B1,B3**) and adenocarcinoma in the left lower lobe (**B2,B4**) of the lung. (**C**) Both of acinar predominant adenocarcinoma in the left upper lobe (**C1,C3**) and lower lobe (**C2,C4**) of the lung. (**D**) Acinar predominant adenocarcinoma in the left lower lobe (**D1,D3**) and lepidic predominant adenocarcinoma in the right lower lobe (**D1,D2**) of the lung. (**E**) Micropapillary predominant adenocarcinoma in the left upper lobe (**E1,E3**) and lepidic predominant adenocarcinoma in the left lower lobe (**E2,E4**) of the lung. (**F**) Papillary predominant adenocarcinoma in the right lower lobe (**F1,F3**) and lepidic predominant adenocarcinoma in the right middle lobe (**F2,F4**) of the lung. (**G**) Solid predominant adenocarcinoma in the upper lower lobe (**G1,G3**) and acinar predominant adenocarcinoma in the left upper lobe (**G2,G4**) of the lung.

During the study period, 290 patients met the modified criteria of Martini and Melamed and were classified with SMPLC. 5 were excluded from data analysis, including 1 patient who died during the perioperative period, 1 who died 45 days postoperatively of respiratory failure after the second operation (0.69%), 2 patients who died from non-cancer-related reasons (1 from acute myocardial infarction and 1 from cerebrovascular accident), and 1 patient who was lost to follow-up. A total of 285 patients were studied in detail to determine prognostic factors (Fig. 1). In this study, long-term survival was defined as 5-years’ cancer-specific survival. Patient demographic characteristics were summarized in Table 1. The median age was 60 years old (range 39–78), and 55.8% of the patients were female. Synchronous multiple primary NSCLCs were detected in a higher proportion of asymptomatic patients (56.1%) and non-smokers (62.8%). A total of 102 patients (35.8%) had a family history of cancer (first-degree relatives: parents, children and sibs). The preoperative (first surgery) serum carcinoembryonic antigen (CEA) level was elevated (≥5.0 ng/mL) in 48 patients (21.9%).

### Surgical treatment and tumor characteristics

Surgical procedures and tumor characteristics were summarized in Table 2. Among the 94 (33.0%) patients with bilateral tumors, a single-stage bilateral operation was performed in 1 patient using VATS, while two-stage bilateral operations were performed in 93 patients; consisting of 48 VATS, 27 thoracotomies and 18 combined surgeries. A total of 87 patients (30.5%) underwent a standard surgical resection called a multi-lobectomy, including 6 pneumonectomies, 37 bilobectomies and 44 lobectomies, for synchronous cancers. Lobectomy combined with sublobar resection (segmentectomy and wedge resection) was performed in 139 patients (48.8%), and 59 patients (20.7%) underwent sublobar resections. The resection margins in all cases were negative.

190 patients (66.7%) had synchronous tumors located in different lobes; 55 patients had tumors located in the same lobe (19.3%); and 40 patients (14.0%) had tumor located in combined lobes (at least 2 tumors within the same lobe,). 234 patients (82.1%) had 2 tumors; 33 patients (11.6%) had 3 tumors, and 18 patients (6.3%) had 4 to 7 tumors. The median size of the maximum diameter of the tumors in each patient was 2.5 cm (range 0.5–7.8 cm), and in 68.8% of the patients it was ≤3 cm. Multiple adenocarcinomas (ADCs) were the predominant histological type, occurring in 233 patients (81.8%). In 9 patients (3.1%), adenocarcinoma was present along with other histological types including adenosquamous carcinoma (n = 4), carcinoid (n = 3), sarcomatoid carcinoma (n = 1) and large cell carcinoma (n = 1). Multiple squamous cell carcinomas (SCCs) were present in 27 patients (9.5%), while ADCs and SCCs occurred simultaneously in 14 patients (4.9%). For multiple ADCs patients, the histologic subtype of the largest tumor were acinar predominant in 122 patients (52.3%), lepidic predominant in 69 patients (29.6%), papillary predominant in 23 patients (9.9%), solid predominant in 13 patients (5.6%) and micropapillary predominant in 6 patients (2.6%). A total of 75 (26.3%) patients were diagnosed with T1 stage, 175 (61.4%) with T2a stage, 15 (5.3%) with T2b stage, and 20 patients (7.0%) with T3-T4 stage tumors. There were 217 patients lymph node-negative (76.1%). Lymph nodes metastases were identified in 68 patients (23.9%), among them, 27 patients had pN1 disease, and 41 had pN2 disease. 119 patients (41.8%) underwent adjuvant chemotherapy treatment that consisted of pemetrexed, gemcitabine, or paclitaxel combined with platinum.

### Clinical Outcomes

The median follow-up period for all 285 patients was 27.6 months (range: 3.2–68.1 months). There were 60 patients (21.1%) who experienced recurrence or distant metastases; and 31 patients (10.9%) were dead at the end of the follow-up period. The 5-year DFS and OS rate was 58.7% and 77.6%, respectively.

Table 3 summarized the associations of the clinicopathological factors with DFS and OS by univariate analysis in all patients. Worse DFS was significantly associated with the following factors: male gender (HR = 1.78, p = 0.025), symptomatic disease (HR = 2.21, p = 0.002), moderate and heavy smoker (p = 0.018), preoperative CEA ≥ 5.0 ng/ml (HR = 1.89, p = 0.032), larger maximal tumor size (p < 0.001), not all ADC (HR = 2.08, p = 0.008), advanced pT (p < 0.001), lymph node involvement (p < 0.001) and adjuvant chemotherapy (HR = 2.07, p = 0.004) (see Supplementary Fig. S1). Worse OS was significantly associated with male gender (HR = 2.27, p = 0.025), symptomatic disease (HR = 3.15, p = 0.002), negative family history of cancer (HR = 2.50, p = 0.038), larger maximal tumor size (p = 0.002), not all ADC (HR = 2.70, p = 0.005), advanced pT (p < 0.001) and lymph node involvement (p < 0.001) (see Supplementary Fig. S2). In the multivariate analysis, after adjusting for the above factors significantly associated with DFS or OS in univariate analysis, only symptomatic disease (HR = 1.89, p = 0.043) and lymph node involvement (p < 0.001) influenced the PFS. For the OS, male gender (HR = 2.56, p = 0.020), symptomatic disease (HR = 2.71, p = 0.017) and lymph node involvement (p < 0.001) were the independent prognosticators for the SMPLC patients (Table 4).

For the 233 patients with multiple lung adenocarcinoma, the 5-year DFS and OS rate was 59.6% and 82.4%, respectively. The univariate analysis showed that symptomatic disease (HR = 2.16, p = 0.011), larger maximal tumor size (p < 0.001), advanced pT (p < 0.001), lymph node involvement (p < 0.001), adjuvant chemotherapy (HR = 2.28, p = 0.008) as well as the subtype other than lepidic predominant (p < 0.001) were significantly associated with the shorter PFS (see Supplementary Fig. S3). The negative family history of cancer (HR = 3.73, p = 0.025), larger maximal tumor size (p = 0.027), advanced pT (p < 0.001), lymph node involvement (p = 0.005) and the subtype other than lepidic predominant (p < 0.001) significantly influenced the OS of patients with multiple lung ADC (see Supplementary Fig. S4). Adjusting for the above factors associated with PFS or OS, the multivariate analysis illustrated that only lymph node involvement (p < 0.001) and the subtype other than lepidic predominant (p = 0.002) were the independent prognosticators for the worse DFS and OS for patients with multiple lung ADC (Tables 5 and 6).

## Discussion

In a retrospective series of 285 patients who underwent surgery for synchronous multiple primary NSCLC, the 5-year DFS and OS rate was 58.7% and 77.6%, respectively, which are comparable to previous reports^10^,^11^,^12^,^13^,^14^,^15^. Multivariate analysis identified symptomatic disease and lymph node involvement as the unfavorable prognostic variables for DFS, while symptomatic disease, lymph node involvement as well as male gender were the independent poor prognosticators for OS in patients with NSCLC. For the 233 patients with multiple lung ADC, lymph node involvement and the subtype other than lepidic predominant were independent predictors for both DFS and OS.

Among the patients who underwent adjuvant chemotherapy treatment, 60 had lymph nodes metastases, while only 8 patients had positive lymph nodes in the non-treated group. The poor DFS observed in the adjuvant chemotherapy group may have been substantially influenced by lymph node involvement rather than treatment. Moreover, univariate analysis showed no difference in OS between the two groups, which are consistent with previous reports suggesting no benefit of postoperative adjuvant treatment^8^,^13^,^16^,^17^. Two groups reported that adjuvant treatment might be favorable in multivariate analyses. However, their conclusion was weakened due to a selection bias^18^ or lack of statistical significant difference in survival^19^. These discrepancies may be attributable to differences in the selection criteria used in different studies, and thus stratified analyses are guaranteed to identify the influential factors. We further analyzed whether adjuvant chemotherapy provided a benefit in the subgroups with or without lymph node involvement. In the negative subgroup, the presence of adjuvant chemotherapy did not significantly affect the 5-year OS rate (85.6% vs 85.0%, p = 0.752). However, in the positive subgroup, patients who received postoperative adjuvant treatment had a much better 5-year OS rate (72.1% vs 0.0%, p = 0.012). Therefore, SMPLC patients with positive lymph node involvement may benefit from postoperative adjuvant chemotherapy treatment.

In the latest revision of T descriptors in the forthcoming 8^th^ TNM classification system for lung cancer, additional pulmonary tumor nodules in the same lobe or in different ipsilateral lobes continue will be categorized as T3 or T4, respectively^2^. However, in our present study, the 5-year OS rate in patients with tumors located in the same lobe was 71.8%, which is much better than the reported 52–56% of T3 stage^2^. Yu *et al*. compared survival between SMPLCs and matched-stage solitary primary lung cancers after surgical treatment, indicating that SMPLCs had an excellent and comparable surgical outcome, which was somewhat discordant with outcomes in T4 or M1 stage patients in the current TNM classification system^8^. In our study, the 5-year OS rate of patients with bilateral tumors was 82.7%, which indicated that it was inappropriate to classify the synchronous multiple primary lung cancers as T3, T4 or M1 stage in the current TNM system.

In the present study, the location of simultaneous tumors was not a prognostic indicator of survival, regardless of the laterality or the distribution of the tumors within the lobe. Similar results have been reported previously, showing no difference in survival according to tumor distribution^8^,^14^,^16^,^17^,^19^,^20^,^21^,^22^. Some studies have argued that having bilateral tumors seems a favorable prognostic indicator^6^,^18^. However, in the study reported by Trousse *et al*.^18^, more patients with unilateral advanced stage diseases were included in the analysis. Tanvetyanon *et al*.^6^ proposed that bilateral tumors had better outcomes mainly based on the literature^23^, but in the referenced study, the authors admitted that they selected more lymph node negative subset in bilateral synchronous tumors, which contributed to the higher 5-year survival rate. Conversely, Ishikawa *et al*. claimed that bilateral tumors predicted poor outcomes^13^, because additional unfavorable factors such larger size of the second tumor and inadequate resection were included. Therefore, with regard to synchronous multiple primary NSCLCs, tumor distribution might not be significantly related to long term survival if selection bias could be strictly controlled.

Tanvetyanon and colleagues observed that adenocarcinoma was independently associated with better outcomes (p < 0.001)^24^, which is in agreement with our results. The 5-year DFS and OS rate for multiple lung ADCs or predominant ADCs in our study was 60.4% and 82.6%, respectively. Lymph node metastases, indicating a more aggressive disease, were confirmed in approximately one-third of the patients with predominant non-ADCs. Regardless of the diagnostic approaches (low-dose CT or chest radiography), lung cancer screening identified the majority of adenocarcinomas, and many cases were detected at the early stages^25^, allowing these patients to benefit from radical surgical resection. It is therefore reasonable that patients without symptomatic disease had better survival than their counterparts.

To the best of our knowledge, we have reported the largest series of surgical outcomes for synchronous multiple primary NSCLCs (Table 7). We found that the independent prognosticators included male gender, symptomatic disease, and lymph node involvement for patients with SMPLC, and lymph node involvement and subtype other than lepidic predominant for patients with multiple lung ADC, which were also identified in previous identical studies^13^,^16^,^18^,^20^,^26^,^27^. Although the Martini and Melamed clinicopathological criteria are widely accepted as a basis for modification and enrichment, remarkable variation is still observed in different reports. The explanations may include the arbitrary nature of modified criteria, selection bias, lack of comprehensive histological assessment or multidisciplinary team revision and an absence of genetic molecular analyses.

In our study, 35.8% of the patients had a family history of cancer in first degree relatives (parents, children and sibs). They had better OS, which has not been described in previous studies. Notwithstanding the fact that the relationship between family history and increased risk of lung cancer is widely accepted, especially in non-smokers^28^, strong evidence and convincing mechanisms for this association remain unidentified. A previous study from our institution reported that patients with a positive family history of lung cancer had a better prognosis (p = 0.015)^29^. Further investigations of synchronous multiple primary NSCLCs may be useful for identifying the genetic factors that predispose individuals to lung cancer.

The strength of our study includes the largest number of enrolled patients with synchronous multiple primary NSCLCs. Modified criteria in the present series enabled a more comprehensive data analysis. However, there are still some limitations in our study. First, it was inevitable that inherent selection bias would be present due to the nature of a single-institution, retrospective study. Second, we did not select patients who underwent incomplete or no surgery, which may have underpowered the ability to identify predictors associated with prognosis. Third, the difficulty we experienced in summarizing the precise TNM stage instead of the pT stage or pN stage in each patient led to an inability to determine the 5-year DFS and OS rate for each stage, which impeded a comparison of stage-matched counterparts.

In summary, for the patients with synchronous multiple primary NSCLC, male gender, symptomatic disease and lymph node involvement were the independent unfavorable prognosticators. In addition, lymph node involvement and subtype other than lepidic predominant were the independent unfavorable prognosticators for patients with multiple lung ADC. Radical surgical resection is feasible and effective treatment for patients with synchronous multiple primary NSCLC, and the identified prognosticators will provide valuable clues for the postoperative management of this population.

## Additional Information

**How to cite this article**: Zhang, Z. *et al*. Surgical Outcomes of Synchronous Multiple Primary Non-Small Cell Lung Cancers. *Sci. Rep.* **6**, 23252; doi: 10.1038/srep23252 (2016).

## Supplementary Material

Supplementary Information

## Figures and Tables

**Table 1:** Patient characteristics.

**Variables**	**Total (n = 285)**
Age, yr, median (range)	60 (39–78)
Sex, n (%)	
Male	126 (44.2)
Female	159 (55.8)
Smoking, n (%)	
Heavy smoker	43 (15.1)
Moderate smoker	44 (15.4)
Light smoker	19 (6.7)
Never	179 (62.8)
Symptom, n (%)	
Fever	6 (2.1)
Dry cough	25 (8.8)
Expectoration	38 (13.3)
Hemoptysis	39 (13.7)
Chest pain	17 (6.0)
No	160 (56.1)
Family history of cancer^a^, n (%)	
Yes	102 (35.8)
No	183 (64.2)
Preoperative CEA level, n (%)	
<5.0 ng/ml	171 (79.1)
≥5.0 ng/ml	48 (21.9)

Abbreviations: CEA, carcinoembryonic antigen;

^a^First degree relatives.

**Table 2:** Surgical and pathological details.

**Variables**	**Total (n = 285)**
Staging operation, n (%)	
Single-stage^a^	192 (67.4)
Two-stage	93 (32.6)
Laterality, n (%)	
Unilateral	191 (67.0)
Bilateral	94 (33.0)
Approach, n (%)	
Unilateral	
Thoracotomy	107 (37.5)
VATS	84 (29.5)
Bilateral	
Thoracotomy+Thoracotomy	27 (9.5)
VATS+VATS^a^	49 (17.2)
VATS+Thoracotomy	18 (6.3)
Type of surgical resection, n (%)	
Multi-lobectomy	87 (30.5)
Lobectomy+sublobar resections^b^	139 (48.8)
Sublobar resections	59 (20.7)
Location of lobe, n (%)	
Same lobe	55 (19.3)
Different lobe	190 (66.7)
Combined lobe^c^	40 (14.0)
No. of tumor, n (%)	
2	234 (82.1)
3	33 (11.6)
≥4	18 (6.3)
Largest T size, cm, n (%)	
≤2	103 (36.2)
2 < d ≤ 3	93 (32.6)
3 < d ≤ 5	72 (25.3)
d > 5	17 (5.9)
Histology type, n (%)	
ADCs (multiple)	233 (81.8)
SCCs (multiple)	27 (9.5)
ADC+other^d^	9 (3.1)
SCC+other^e^	2 (0.7)
ADC+SCC	14 (4.9)
Subtypes of largest tumor^f^, n (%)	
Lepidic predominant	69 (29.6)
Acinar predominant	122 (52.3)
Papillary predominant	23 (9.9)
Micropapillary predominant	6 (2.6)
Solid predominant	13 (5.6)
Highest pT stage^g^, n (%)	
1a	11 (3.8)
1b	45 (15.8)
1c	19 (6.7)
2a^h^	175 (61.4)
2b	15 (5.3)
3	16 (5.6)
4	4 (1.4)
pN stage, n (%)	
0	217 (76.1)
1	27 (9.5)
2	41 (14.4)
Adjuvant chemotherapy^i^, n (%)	
Yes	119 (41.8)
No	166 (58.2)

Abbreviations: VATS, video-assisted thoracic surgery. ADC, adenocarcinoma. SCC, squamous cell carcinoma.

^a^One was single-stage treatment of bilateral Cancers via VATS.

^b^Segmentectomy and wedge resection.

^c^More than 2 cancers, at least 2 tumors were located at the same lobe and the other or others located at the different.

^d^Adenosquamous carcinoma, carcinoid, sarcomatoid carcinoma and large cell carcinoma.

^e^Pleomorphic carcinoma and carcinoid.

^f^Multiple adenocarcinomas (n = 233).

^g^The new revision of T stage in the forthcoming 8^th^ TNM system.

^h^Including a tumor with a diameter ≤3 cm but invades visceral pleura.

^i^Pemetrexed, gemcitabine, or paclitaxel combined with platinum.

**Table 3:** Univariate analysis of predictors for 5-year disease free survival and overall survival rates in patients with SMPLC.

**Variables**	**Disease free survival**			**Overall survival**		
	**HR**	**95% CI**	***P*** **value**	**HR**	**95% CI**	***P*****value**
Age, yrs			0.450			0.269
<60	1.00	Reference		1.00	Reference	
≥60	1.22	0.73–2.04		1.51	0.72–3.15	
Sex			0.025			0.025
Female	1.00	Reference		1.00	Reference	
Male	1.78	1.07–2.97		2.27	1.08–4.73	
Symptoms^a^			0.002			0.002
No	1.00	Reference		1.00	Reference	
Yes	2.21	1.31–3.72		3.15	1.45–6.85	
Smoking			0.018			0.096
No	1.00	Reference		1.00	Reference	
Light smoker	0.70	0.17–2.93		0.84	0.11–6.42	
Moderate smoker	2.08	1.09–4.00		2.63	1.10–6.27	
Heavy smoker	2.21	1.18–4.11		2.06	0.87–4.93	
Family history of cancer^b^			0.208			0.038
Yes	1.00	Reference		1.00	Reference	
No	1.43	0.82–2.48		2.50	1.02–6.10	
Preoperative CEA level			0.032			0.275
<5.0 ng/ml	1.00	Reference		1.00	Reference	
≥5.0 ng/ml	1.89	1.05–3.42		1.55	0.70–3.40	
Laterality			0.504			0.219
Unilateral	1.00	Reference		1.00	Reference	
Bilateral	0.84	0.49–1.42		0.62	0.29–1.33	
Lobe			0.505			0.933
Same lobe	1.00	Reference		1.00	Reference	
Different lobe	1.57	0.71–3.49		1.08	0.41–2.85	
Combined^c^	1.68	0.61–4.63		0.87	0.21–3.66	
Type of resection			0.288			0.904
Multi-lobectomys	1.00	Reference		1.00	Reference	
Lobectomy+sublobar resections^d^	1.52	0.83–2.79		1.00	0.44–2.30	
Sublobar resections	1.02	0.46–2.28		1.22	0.45–3.27	
No. of tumor			0.645			0.510
2	1.00	Reference		1.00	Reference	
≥3	0.85	0.42–1.72		0.70	0.25–2.01	
Largest T size, cm			<0.001			0.002
d ≤ 2	1.00	Reference		1.00	Reference	
2 < d ≤ 3	2.33	1.07–5.09		2.24	0.70–7.13	
3 < d ≤ 5	3.71	1.71–8.06		3.85	1.22–12.09	
d > 5	5.83	2.24–15.21		8.55	2.40–30.54	
Histology type			0.008			0.005
All ADCs	1.00	Reference		1.00	Reference	
Not all ADCs	2.08	1.19–3.63		2.70	1.31–5.59	
Highest pT stage			<0.001			<0.001
1^e^	1.00	Reference		1.00	Reference	
2a	14.70	2.02–106.83		–	–	
2b	30.76	3.59–263.51		–	–	
3+4	37.55	4.80–294.05		–	–	
pN stage			<0.001	–		<0.001
0	1.00	Reference		1.00	Reference	
1	3.25	1.62–6.52		3.28	1.26–8.55	
2	5.51	3.12–9.75		5.07	2.30–11.20	
Adjuvant chemotherapy^f^			0.004			0.169
No	1.00	Reference		1.00	Reference	
Yes^g^	2.07	1.24–3.45		1.63	0.81–3.30	

HR, Hazard ratio; CI, confidence interval; NSCLC, non-small cell lung cancer; ADC, adenocarcinoma; SCC, squamous cell cancer; CEA, carcinoembryonic antigen; pT, tumor; pN, lymph node; d, maximum diameter.

^a^Including fever, cough, expectoration, hemoptysis, and chest pain.

^b^First degree relatives.

^c^More than 2 cancers, at least 2 tumors were located at the same lobe and the other or others located at the different.

^d^Segmentectomy and wedge resection.

^e^No patient with T1 stage was dead.

^f^Pemetrexed or gemcitabine or paclitaxel combined with platinum.

^g^Including 119 patients, among them, 60 had lymph nodes metastases.

**Table 4:** Multivariable analysis of survival predictors for patients with SMPLC.

**Variables**	**Disease free survival**			**Overall survival**		
	**HR**	**95% CI**	***P*****value**	**HR**	**95% CI**	***P*** **value**
Sex			0.249			0.020
Female	1.00	Reference		1.00	Reference	
Male	1.49	0.76–2.94		2.56	1.16–5.68	
Symptoms^a^			0.043			0.017
No	1.00	Reference		1.00	Reference	
Yes	1.89	1.02–3.51		2.71	1.19–6.14	
pN stage			<0.001			<0.001
0	1.00	Reference		1.00	Reference	
1	1.63	0.73–3.66		2.33	0.88–6.15	
2	2.07	1.10–3.88		5.48	2.36–12.74	

HR, Hazalrd ratio; CI, confidence interval.

^a^Including fever, cough, expectoration, hemoptysis, and chest pain.

**Table 5:** Univariate analysis of predictors for 5-year disease free survival and overall survival rates in patients with synchronous multiple lung adenocarcinoma.

**Variables**	**Disease free survival**			**Overall survival**		
	**HR**	**95% CI**	***P*** **value**	**HR**	**95% CI**	***P*** **value**
Age, yrs			0.876			0.966
<60	1.00	Reference		1.00	Reference	
≥60	0.95	0.52–1.75		1.02	0.41–2.51	
Sex			0.384			0.289
Female	1.00	Reference		1.00	Reference	
Male	1.32	0.71–2.45		1.63	0.66–4.01	
Symptom^a^			0.011			0.055
No	1.00	Reference		1.00	Reference	
Yes	2.16	1.17–3.97		2.37	0.95–5.90	
Smoking			0.139			0.283
No	1.00	Reference		1.00	Reference	
Light smoker^b^	–	–		–	–	
Moderate smoker	2.46	1.16–5.21		2.58	0.92–7.24	
Heavy smoker	1.19	0.42–3.40		0.60	0.08–4.57	
Family history of cancer^c^			0.275			0.025
Yes	1.00	Reference		1.00	Reference	
No	1.43	0.75–2.72		3.73	1.09–12.85	
Preoperative CEA level			0.147			0.163
<5.0 ng/ml	1.00	Reference		1.00	Reference	
≥5.0 ng/ml	1.75	0.82–3.74		2.03	0.75–5.51	
Laterality			0.638			0.287
Unilateral	1.00	Reference		1.00	Reference	
Bilateral	0.86	0.46–1.61		0.59	0.22–1.56	
Lobe			0.335			0.658
Same lobe	1.00	Reference		1.00	Reference	
Different lobe	1.99	0.70–5.65		1.87	0.42–8.25	
Combined^d^	2.48	0.72–8.49		2.21	0.37–13.35	
Type of resection			0.456			0.535
Multi-lobectomys	1.00	Reference		1.00	Reference	
Lobectomy+sublobar resections^e^	1.53	0.73–3.22		0.99	0.32–3.06	
Sublobar resections	1.09	0.43–2.77		1.74	0.53–5.71	
No. of tumor			0.985			0.826
2	1.00	Reference		1.00	Reference	
≥3	1.01	0.47–2.18		1.13	0.38–3.42	
Largest T size, cm			<0.001			0.027
d ≤ 2	1.00	Reference		1.00	Reference	
2<d ≤ 3	3.08	1.21–7.81		2.23	0.57–8.63	
3<d ≤ 5	4.45	1.74–11.39		3.65	0.94–14.14	
d > 5	11.85	2.92–48.05		10.44	1.74–62.55	
Subtypes of largest tumor			<0.001			<0.001
Lepidic predominant	1.00	Reference		1.00	Reference	
Acinar predominant	4.92	1.59–15.24		8.33	1.03–67.42	
Papillary predominant	6.84	1.84–25.40		3.92	0.25–62.85	
Micropapillary predominant	8.47	1.89–37.88		9.22	0.58–147.94	
Solid predominant	24.69	7.11–85.70		57.02	7.07–459.69	
Highest pT stage			<0.001			<0.001
1^f^	1.00	Reference		1.00	Reference	
2a	–	–		–	–	
2b	–	–		–	–	
3+4	–	–		–	–	
pN stage			<0.001			0.005
0	1.00	Reference		1.00	Reference	
1	3.73	1.56–8.89		3.03	0.82–11.21	
2	6.24	3.19–12.21		4.94	1.83–13.28	
Adjuvant chemotherapy^g^			0.008			0.186
No	1.00	Reference		1.00	Reference	
Yes^h^	2.28	1.24–4.21		1.84	0.75–4.53	

HR, Hazard ratio; CI, confidence interval; ADC, adenocarcinoma; SCC, squamous cell cancer; CEA, carcinoembryonic antigen; pT, tumor; pN, lymph node; d, maximum diameter.

^a^Including fever, cough, expectoration, hemoptysis, and chest pain.

^b^No light smoker was relapsed or dead.

^c^First degree relatives.

^d^More than 2 cancers, at least 2 tumors were located at the same lobe and the other or others located at the different.

^e^Segmentectomy and wedge resection.

^f^No patient with T1 stage was relapsed or dead.

^g^Pemetrexed or gemcitabine or paclitaxel combined with platinum.

^h^Including 91 patients, but 47 had lymph nodes metastases.

**Table 6:** Multivariable analysis of survival predictors for patients with synchronous multiple lung adenocarcinoma.

**Variables**	**Disease free survival**			**Overall survival**		
	**HR**	**95% CI**	***P*****value**	**HR**	**95% CI**	***P*** **value**
Subtypes of largest tumor			<0.001			<0.001
Lepidic predominant	1.00	Reference		1.00	Reference	
Acinar predominant	3.18	0.89–11.33		6.36	0.76–53.57	
Papillary predominant	2.72	0.54–13.65		2.79	0.17–45.21	
Micropapillary predominant	25.16	2.83–223.71		25.56	1.10–595.43	
Solid predominant	23.41	5.75–95.24		51.57	5.85–454.64	
pN stage			<0.001			0.002
0	1.00	Reference		1.00	Reference	
1	1.36	0.39–4.76		1.06	0.22–5.03	
2	6.67	2.91–15.32		7.41	2.35–23.43	

HR, Hazard ratio; CI, confidence interval; pN, lymph node.

**Table 7:** Summary of the previous major series on surgical treatment for SMPLCs and the statistically significant prognosticators using multivariate analysis over the last 15 years.

**Authors**	**Time**	**Reference**	**No.**	**OS rate (%)**		**Variables**	**HR (95% CI)**	***P*** **value**
				**3-yr**	**5-yr**			
Roberts *et al*.	2003	21	14	NR	64^a^	–	–	–
Nakata *et al*.	2004	10	26	92.9^b^	NR	–	–	–
Tsunezuka *et al*.	2004	11	18^c^	–	69	–	–	–
Mun *et al*.	2007	12	19	94.7	75.8	–	–	–
Chang *et al*.	2007	26	92	–	35.3	Lymph node metastasis	2.37 (1.20–4.69)	0.013
Trousse *et al*.	2007	18	125	–	34	Sex (male vs female)	2.50 (1.15–5.42)	0.021
						Age (<60y vs ≥60y)	0.53 (0.31–0.91)	0.022
						Pneumonectomy (yes vs. no)	6.60 (3.34–13.1)	<0.0001
						FEV_1_ (%) (high vs. low)	0.95 (0.93–0.98)	<0.0001
						Asymptomatic (no vs. yes)	0.42 (0.24–0.72)	0.002
						Optimal surgery (no vs. yes)	2.30 (1.14–4.75)	0.02
						Adjuvant treatment (no vs. yes)	1.80 (1.02–3.33)	0.043
						pN0 disease (no vs. yes)	2.90 (1.20–6.90)	0.004
Riquet *et al*.	2008	23	118	–	26	–	–	–
Rostad *et al*.	2008	27	94	–	27.6	Male	1.70 (1.01–2.86)	0.044
						Age > 70y	1.91 (1.15–3.19)	0.013
						Pneumonectomy	1.96 (1.18–3.26)	0.009
						Adenocarcinoma	2.15 (1.09–4.24)	0.053
Finley *et al*.	2010	20	175	64	–	Male	2.21 (1.45–3.38)	<0.001
Tanvetyanon *et al*.	2010	22	116	–	–	FEV_1_ (%) (<80% vs. ≥80%)	2.03 (1.07–3.83)	0.01
						Largest tumor size	1.17 (1.06–1.30)	0.01
						Sum of tumor sizes	1.15 (1.05–1.26)	0.01
Voltolini *et al*.	2010	16	43	NR	34	N stage (pN0 vs. pN1–2)	0.20 (0.08–0.55)^d^	0.002
						Period (1990–99 vs. 2000–07)	4.22 (1.74–10.4)^d^	0.001
Fabian *et al*.	2011	14	67	73^e^	69^e^	–	–	–
Kocaturk *et al*.	2011	19	26	–	49.7	Pneumonectomy (yes vs. no)	1.68 (0.96–30.2)	0.05^f^
						Adjuvant treatment (no vs. yes)	1.80 (0.88–41.7)	0.06^f^
Jung *et al*.	2011	17	32	–	60.9	Not identified		
Yu *et al*.	2013	8	97	83.1	69.6	Lymphovascular invasion	3.94 (no detail)	0.004
						Tumor size	2.92 (no detail)	0.036
Ishikawa et al	2014	13	93^g^	–	87.0	Distribution (bilateral vs. ipsilateral)	4.63 (1.15–18.7)	0.031
						Lymph node involvement (yes vs. no)	10.6 (2.14–52.1)	0.004
						Sublobar resection (yes vs. no)	4.43 (1.05–18.6)	0.042
Shimada *et al*.	2015	15	67^h^	–	53.6–95.8^i^	Size of main cancer	3.97 (1.33–11.9)	0.049

OS, overall survival; HR, hazards ratio; CI, confidence interval.

^a^Multifocal bronchioloalveolar carcinoma.

^b^Including 5 five patients who were metachronous without disease recurrences.

^c^Bilateral multiple primary lung cancers.

^d^relative ratio value, not HR.

^e^Cancer-specific survival rate.

^f^A trend.

^g^Synchronous primary lung adenocarcinomas.

^h^Lung cancer patients with synchronous multiple ground-glass opacities.

^i^Different groups.
