# Supplementary material for: Surgical Outcomes of Synchronous Multiple Primary Non-Small Cell Lung Cancers
Source: Sci Rep. 2016 Jun 2;6:23252. doi: 10.1038/srep23252 (PMC4890551; doi:10.1038/srep23252)
Supplement: Supplementary Information [file srep23252-s1.docx]

**Title:** Surgical Outcomes of Synchronous Multiple Primary Non-Small Cell Lung Cancers

**Authors:** Zhirong Zhang, Shugeng Gao, Yousheng Mao, Juwei Mu, Qi Xue,

Xiaoli Feng, Jie He.


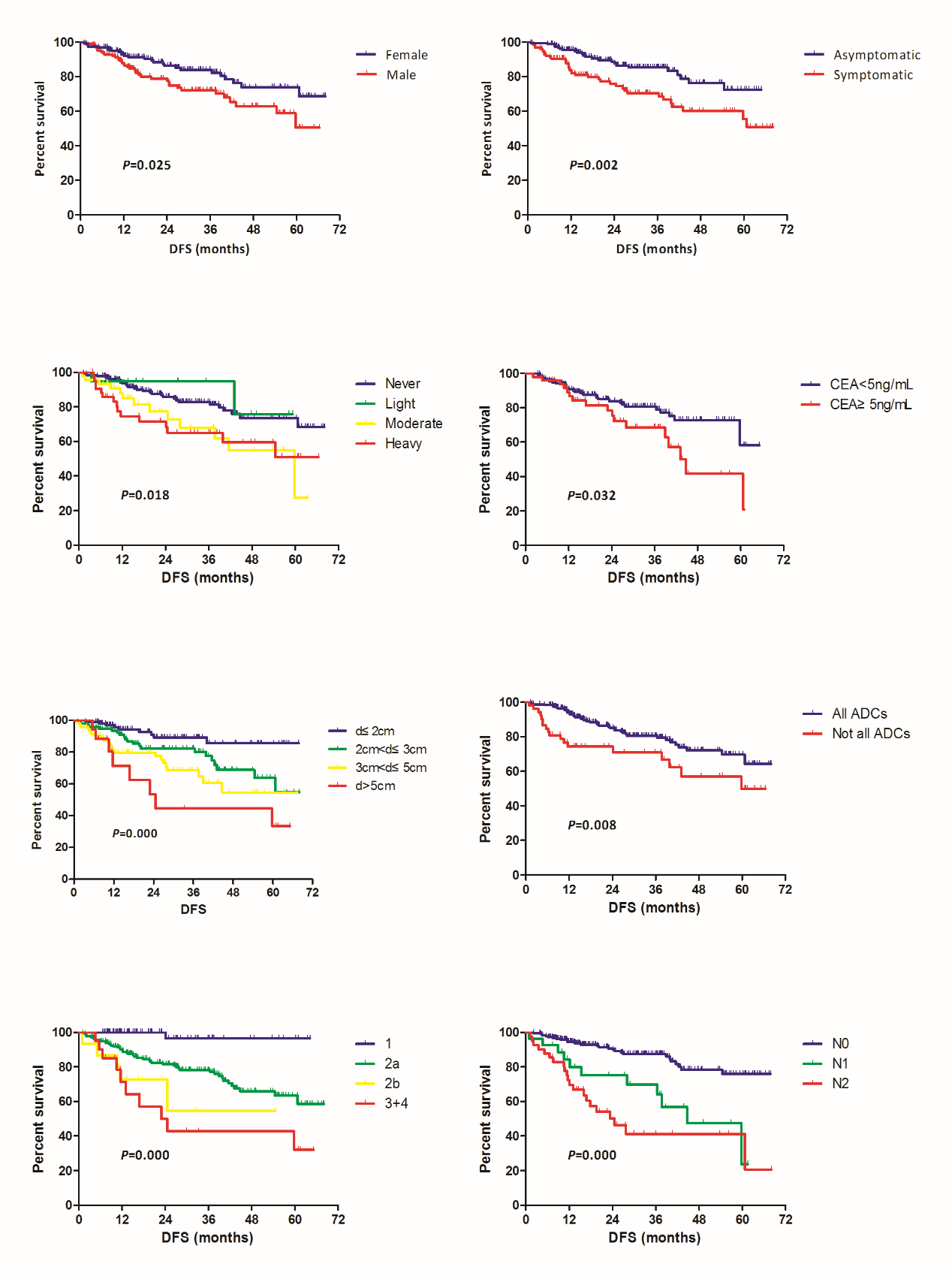


**Supplementary Figure S1.** DFS by gender, symptom status, smoking, CEA level, largest T size, histology type, highest T stage and lymph nodes status in patients with SMPLC using Kaplan-Meier method. ADC, adenocarcinoma.


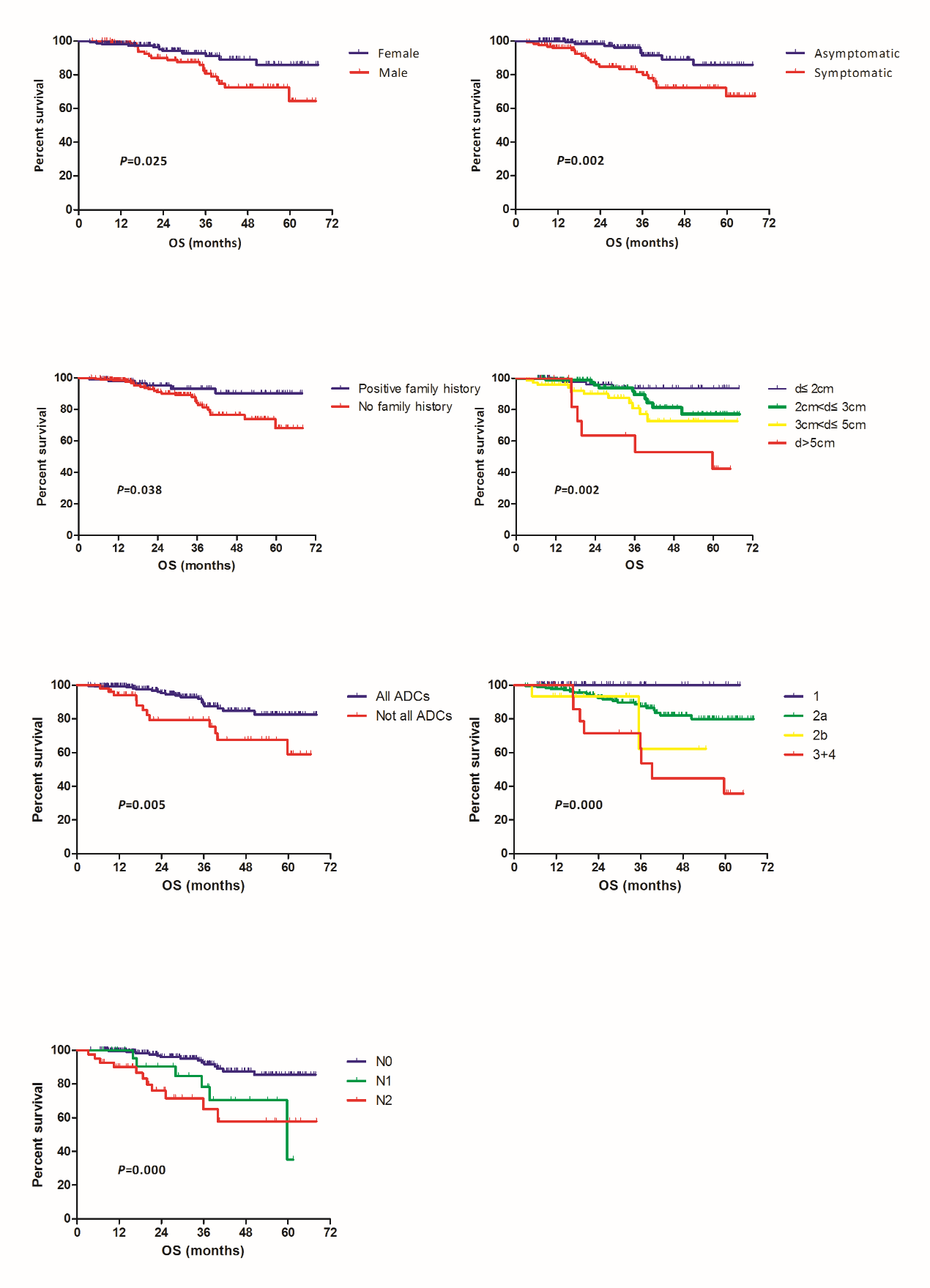


**Supplementary Figure S2.** OS by gender, symptom status, family history of cancer, largest T size, histology type, highest T stage and lymph nodes status in patients with SMPLC using Kaplan-Meier method. ADC, adenocarcinoma.


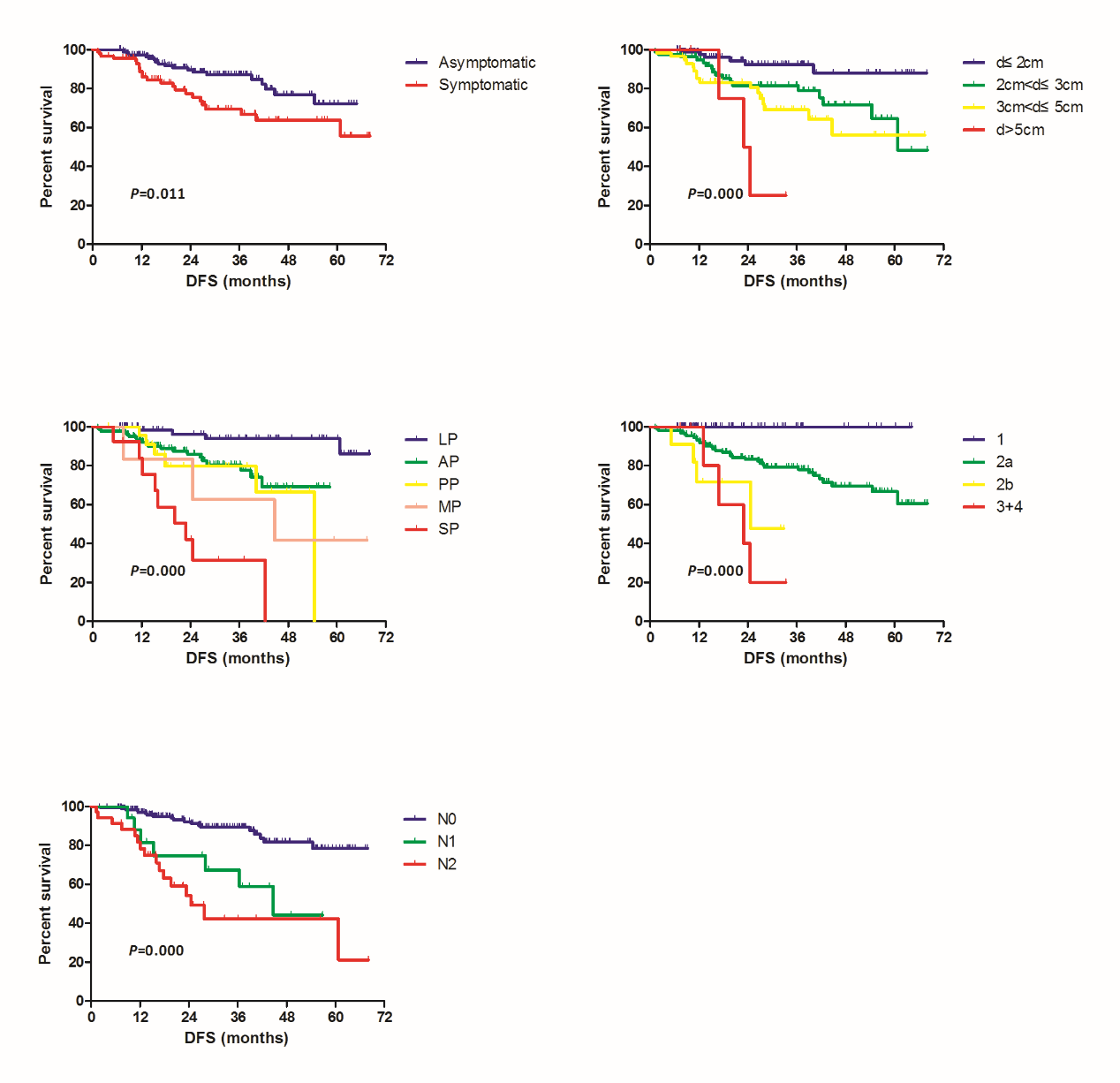


**Supplementary Figure S3.** DFS by symptom status, largest T size, predominant subtype of largest tumor, highest T stage and lymph nodes status in patients with synchronous multiple lung adenocarcinoma using Kaplan-Meier method. LP, lepidic predominant; AP, acinar predominant; PP, papillary predominant; MP, micropapillary predominant; SP, solid predominant.





**Supplementary Figure S4.** OS by family history of cancer, largest T size, predominant subtype of largest tumor, highest T stage and lymph nodes status in patients with synchronous multiple adenocarcinoma using Kaplan-Meier method. LP, lepidic predominant; AP, acinar predominant; PP, papillary predominant; MP, micropapillary predominant; SP, solid predominant.
